# A Delayed Adverse Reaction: Hydralazine-Induced Lupus After Years of Use

**DOI:** 10.7759/cureus.87514

**Published:** 2025-07-08

**Authors:** Saurav Paudel, Ayushma Acharya, Sanjiv Poudel, Swarup Sharma Rijal

**Affiliations:** 1 Internal Medicine, Kist Medical College, Kathmandu, NPL; 2 Internal Medicine, Tower Health Reading Hospital, Reading, USA; 3 Internal Medicine, Lakecity Hospital and Critical Care Pvt Ltd., Pokhara, NPL

**Keywords:** anti-histone antibodies, case report rheumatology, drug-induced-lupus, hydralazine-induced lupus syndrome, hydralazine toxicity

## Abstract

Drug-induced lupus (DIL) is a rare autoimmune condition triggered by certain medications, most commonly hydralazine and procainamide. DIL can manifest after a few weeks or even after several years of drug exposure. We present a case of a 61-year-old female with hypertension and type 2 diabetes mellitus who developed progressive polyarthritis, myalgia, and fatigue that developed over a year after three years of hydralazine use. Laboratory findings revealed anemia, leukopenia, elevated inflammatory markers, an anti-nuclear antibody (ANA) titer of 1:2560, and anti-histone antibodies of 4.9 units. A false-positive immunoglobulin M (IgM) Lyme antibody complicated the diagnostic workup. Discontinuation of hydralazine and initiation of corticosteroid and methotrexate resulted in resolution after six weeks, confirming the diagnosis of hydralazine-induced lupus.

## Introduction

Drug-induced lupus (DIL) is an autoimmune phenomenon characterized by symptoms resembling those of systemic lupus erythematosus (SLE) after exposure to certain drugs. Commonly implicated drugs include hydralazine, procainamide, quinidine, methyldopa, isoniazid, minocycline, and tumor necrosis factor inhibitors like etanercept and infliximab [1]. Rarer incidences have been reported with the use of many other drugs, too. Among implicated medications, procainamide and hydralazine are reported to have the highest incidence, with risks reported as high as 30% for procainamide and 5-10% for hydralazine [1]. DIL constitutes 6-12% of all lupus cases, and the annual incidence is estimated to be between 15,000 and 30,000 new cases in the United States [2].

DIL is characterized by arthralgias or arthritis, myalgias, and rashes, with rarer cases having renal and central nervous system involvement. The onset of DIL is variable, with symptoms developing anywhere from several weeks to several years after initiation of the offending agent [3].

## Case presentation

A 61-year-old female with a past medical history of essential hypertension and type 2 diabetes mellitus presented to her primary care physician with mild joint pain persisting for approximately 12 months, which had demonstrated a progressive worsening over the past four to five weeks with associated visible joint swelling. Symptoms primarily affected the small joints of the bilateral hands, wrists, and the left knee. She complained of associated fatigue, myalgia, and difficulty with ambulation due to pain. The joint pain was worse in the afternoon and evening. She denied morning stiffness, photosensitive rash, skin thickening, oral ulcers, chest pain, dyspnea, or unintentional weight loss. The patient had been using hydralazine 100 mg twice daily for three years. Her other medications included hydrochlorothiazide, valsartan, and carvedilol. Hydrochlorothiazide, in particular, was started only three weeks before the patient’s presentation.

At the time of presentation, the patient’s blood pressure was 137/86 mmHg, temperature was 36.5°C, respiratory rate was 16 breaths/min, pulse rate was 85/min, and oxygen saturation was 97% in room air. Physical examination was significant for swelling and tenderness in all metacarpophalangeal (MCP) joints bilaterally, worse in the first and second MCP joints.

Complete blood count (CBC) was significant for hemoglobin of 10.1 g/dL and white blood cell count (WBC) of 2,100/µl. Anti-nuclear antibody (ANA) was 1:2560 in a homogenous pattern, anti-double-stranded deoxyribonucleic acid (anti-dsDNA) antibody was 1:160, anti-histone antibody was 4.9 units, C-reactive protein (CRP) was 4.14 mg/dL, and erythrocyte sedimentation rate (ESR) was 47 mm/h. X-rays of bilateral hands were taken, which revealed mild degenerative changes, but these were considered to be incidental findings. 

Our patient also tested positive for immunoglobulin M (IgM) Lyme antibody; however, this was considered a false positive, considering the chronic nature of symptoms persisting for approximately a year, which was inconsistent with acute Lyme disease. Additionally, there was no reported history of tick bite or potential exposure (Table 1).

**Table 1:** Laboratory results IgM, immunoglobulin M; IgG, immunoglobulin G; anti-dsDNA, anti-double-stranded deoxyribonucleic acid; anti-SSA, anti-Sjögren’s syndrome type A; anti-SSB, anti-Sjögren’s syndrome type B; anti-RNP, anti-ribonucleoprotein; PTH, parathyroid hormone; TSH, thyroid-stimulating hormone

Test (units)	Result	Reference range
Anti-nuclear antibody	>1:2560 (homogenous pattern)	<1:80
C-reactive protein (mg/dL)	4.14	<1.00
Erythrocyte sedimentation rate (mm/h)	47	0-20
Rheumatoid factor (IU/mL)	<10.0	≤14
White blood cell count (cells/µL)	2100	4800-10,800
Hemoglobin (g/dL)	10.1	12.0-16.0
Platelets (cells/µL)	398,000	130,000-400,000
Lyme IgM antibody	Positive	Negative
Lyme IgG antibody	Negative	Negative
Uric acid (mg/dL)	4.2	2.3-6.6
Anti-dsDNA antibody	1:160	<1:10
Histone IgG antibody (units)	4.9	0.0-0.9
Cyclic citrullinated peptide antibody (U/mL)	<0.5	<5.0
SSA (Ro) IgG antibody (AU/mL)	0	<29
SSB (La) IgG antibody (AU/mL)	0	<29
Anti-RNP antibody (units)	3	<19
Anti-Smith antibody (AU/mL)	2	0-40
PTH (pg/mL)	19	12-88
TSH (mIU/mL)	3.234	0.450-5.330
Vitamin D (ng/mL)	22.6	>20
Hepatitis C antibody	Non-reactive	Non-reactive

Her symptoms, history of hydralazine use, laboratory findings of anemia, leukopenia, elevated inflammatory markers, and positive autoantibodies, especially anti-histone and ANA, were all diagnostic of DIL. After discontinuing hydralazine, the patient was started on naproxen and oxycodone/paracetamol, but due to inadequate relief of symptoms even after three weeks, the patient was started on prednisone as well. A decision was made to initiate methotrexate after a week of initiating steroids due to incomplete resolution. This combination of drugs, along with discontinuation of hydralazine, resulted in complete resolution of symptoms after six weeks.

## Discussion

Hydralazine-induced lupus occurs in fewer than 10% of patients receiving the medication, with the risk increasing in a dose-dependent manner [3]. Hydralazine has been traditionally used as an antihypertensive agent, and more recently, its use has increased due to evidence supporting its ability to reduce mortality and morbidity in heart failure, particularly among African-American populations [4,5].

Clinical manifestations of hydralazine-induced lupus can vary significantly among individuals, but arthralgia, arthritis, or myalgia are noted in approximately 80% of patients affected by hydralazine-induced lupus [6]. Constitutional symptoms, including fever, weight loss, and fatigue, are reported in 40-50%, while rash is reported in about 25%. Hematological abnormalities, including anemia and leukopenia, are observed in 35% and 5-25% of cases, respectively [6]. Clinical manifestations also seem to vary depending on which offending drug is used. For instance, pleuritis and pleural effusion are observed more frequently in procainamide-induced lupus (44%) compared to hydralazine-induced lupus (5%) [6]. Anti-dsDNA antibody, which is present in 50-70% of SLE, is seen in <5% of DIL cases [6]. In contrast, ANA is positive in 95-100% of patients with DIL, although rare instances of ANA-negative DIL have also been reported [7]. One notable thing to consider in DIL is the presence of vasculitis, particularly anti-neutrophil cytoplasmic antibodies (ANCA)-associated vasculitis, as it often manifests with more severe symptoms resulting from multi-organ involvement, including glomerulonephritis, alveolar hemorrhage, and systemic vasculitis [8].

The diagnosis of DIL in our patient was supported by the clinical presentation, history of hydralazine use, laboratory findings of anemia and leukopenia, elevated inflammatory markers, and presence of autoantibodies, specifically positive anti-histone and ANA. A notable finding in our patient was the presence of a positive IgM Lyme antibody. Lyme disease has been reported as a potential trigger for autoimmune conditions, including SLE [9], but treating the condition would have required anti-Lyme antibiotics, which we did not use in our patient. Furthermore, Lyme antibody has also been known to be falsely positive in SLE and other autoimmune conditions [10], which might have been the case in our patient as well.

We were able to rule out valsartan and carvedilol as potential triggers of DIL, as neither has been associated with this condition in the literature. Additionally, hydrochlorothiazide was initiated only three weeks prior to the patient’s initial presentation, making it an unlikely causative agent.

Early recognition and management of DIL are crucial to prevent rare but potentially severe complications, including diffuse alveolar hemorrhage [11], lupus nephritis [12], and pericardial effusion [7]. DIL typically improves with discontinuation of the offending medication; however, some patients, such as ours, may require treatment with corticosteroids and immunosuppressive therapy to achieve clinical resolution.

## Conclusions

This case report highlights the clinical presentation of an uncommon but important condition. Hydralazine is one of the most commonly implicated agents in DIL. Clinical manifestations are variable, thus necessitating a high index of suspicion when SLE-like features are encountered in patients taking lupus-inducing medications. While no standardized diagnostic criteria have been established, the presence of positive ANA and anti-histone antibodies, along with SLE-like symptoms, particularly arthralgia, arthritis, or myalgia, in patients receiving known lupus-inducing medications such as hydralazine, is often sufficient to support a diagnosis of DIL. Resolution of symptoms following cessation of the offending drug is common, yet not absolute in every case. Use of immunosuppressants and corticosteroids can help achieve proper resolution of symptoms. Monitoring of the recurrence of symptoms is necessary to ensure a complete cure. Additionally, periodic autoantibody screening or early evaluation of emerging symptoms of DIL, or both, in patients receiving drugs implicated in DIL may aid in early detection and timely intervention.
